# Supplementary material for: School Students’ Concerns and Support after One Year of COVID-19 in Austria: A Qualitative Study Using Content Analysis
Source: Healthcare (Basel). 2022 Jul 18;10(7):1334. doi: 10.3390/healthcare10071334 (PMC9315779; doi:10.3390/healthcare10071334)
Supplement: Supplementary file 1 [file healthcare-10-01334-s001.zip › healthcare-1817118-supplementary.pdf]

Category system that emerged from the data for Question 1: "What currently gives you most cause for concern?"

	N	%
**School-related concerns**	135	63,1%
Stress at school	93	43,5%
School organisation	19	8,9%
Graduation	17	7,9%
Teachers	6	2,8%
**Restrictions**	75	35,0%
Restrictions of public life/home confinement	29	13,6%
Lack of activities with friends	21	9,8%
Lack of social contacts	13	6,1%
Lack of exercise	5	2,3%
Travel restrictions	4	1,9%
Quarantine	3	1,4%
**Self-related concerns**	68	31,8%
Concerns about the future	22	10,3%
Negative thoughts and emotions	13	6,1%
Lack of drive	8	3,7%
Missing out on something	7	3,3%
Mental health stresses	6	2,8%
Physical health stresses	6	2,8%
Loneliness and social isolation	3	1,4%
Body-related worries	3	1,4%
**Family, relationship and interpersonal problems**	27	12,6%
Family problems	9	4,2%
Relationship troubles	7	3,3%
Relational problems with others	6	2,8%
Worrying about others	5	2,3%
**The pandemic**	25	11,7%
General statements	12	5,6%
Uncertainty of the future	13	6,1%
**Current societal developement**	11	5,1%
**Other concerns**	11	5,1%
**Consumption**	5	2,3%
Alcohol	1	0,5%
Drugs	1	0,5%
Mobile phone/laptop usage	2	0,9%
Sex	1	0,5%
**No stressors**	7	3,3%

Category system that emerged from the data for Question 2: "What is currently providing you with the most support?"

		N	%
**Social contacts**		153	71,5%
	Friends	64	29,9%
	Family	36	16,8%
	Partner	21	9,8%
	Talking to someone	19	8,9%
	Classmates	6	2,8%
	Best friend	4	1,9%
	Other people	3	1,4%
**Recreational activities**		84	39,3%
	Sports	30	14,0%
	Listening to music	24	11,2%
	Going for a walk	9	4,2%
	Hobbies (general category)	8	3,7%
	Relaxation	8	3,7%
	Reading	5	2,3%
**Attitudes and abilities**		39	18,2%
	Mental abilities	30	14,0%
	Structure	5	2,3%
	Abreact	4	1,9%
**Distraction**		36	16,8%
	Gaming	10	4,7%
	Television	8	3,7%
	Social Media Activities	6	2,8%
	Surfing the internet	5	2,3%
	Distraction (general category)	5	2,3%
	Shopping	2	0,9%
**Escape**		23	10,7%
	Sleeping	12	5,6%
	Eating	6	2,8%
	Substances	5	2,3%
**Creativity**		10	4,7%
	Writing/Drawing	7	3,3%
	Making music	3	1,4%
**Professional help**		7	3,3%
**Pets**		5	2,3%
**Faith**		3	1,4%
**School as a resource**		3	1,4%
I don`t know		3	1,4%
Nothing		8	3,7%
I don`t need help		3	1,4%
